# Momentum-resolved spectroscopy of a Fermi liquid

**DOI:** 10.1038/srep09539

**Published:** 2015-05-05

**Authors:** Elmer V. H. Doggen, Jami J. Kinnunen

**Affiliations:** 1 COMP Centre of Excellence and Department of Applied Physics, Aalto University, FI-00076 Aalto, Finland

## Abstract

We consider a recent momentum-resolved radio-frequency spectroscopy experiment, in which Fermi liquid properties of a strongly interacting atomic Fermi gas were studied. Here we show that by extending the Brueckner-Goldstone model, we can formulate a theory that goes beyond basic mean-field theories and that can be used for studying spectroscopies of dilute atomic gases in the strongly interacting regime. The model hosts well-defined quasiparticles and works across a wide range of temperatures and interaction strengths. The theory provides excellent qualitative agreement with the experiment. Comparing the predictions of the present theory with the mean-field Bardeen-Cooper-Schrieffer theory yields insights into the role of pair correlations, Tan's contact, and the Hartree mean-field energy shift.

Strongly interacting fermionic systems are ubiquitous in nature; they are found from solid state systems^1^ and fermionic superfluids to neutron stars and nuclear matter. In the context of ultracold atoms, the transition from weak to strong interactions is described by the crossover from Bardeen-Cooper-Schrieffer (BCS) theory to a Bose-Einstein condensate (BEC) of pairs of fermions^2^. In between these two regimes of weakly interacting particles, the system is in the *unitary* regime^3^, where the absence of a small parameter makes standard perturbation theory inadequate. These systems are therefore more difficult to describe theoretically. In the highly controllable environment of ultracold atoms, one can tune the interactions using Feshbach resonances^4^, making the BCS-BEC crossover accessible in the experiment.

On the BCS side of the crossover, the system is found in a superfluid state below a certain critical temperature *T*_c_, where BCS theory is applicable. In this state, fermions form so-called Cooper pairs in momentum space. Above *T*_c_, in the *normal state*, the pairs are not formed, and the system is found to be described well as a Fermi liquid^5^. In a Fermi liquid, the system behaves similar to a non-interacting gas of fermions, with well-defined and long-lived fermionic quasiparticles which have an effective mass. In this phase, the momentum distribution has a “jump” of size *Z* at the Fermi momentum *k*_F_. The value of *Z*, the *quasiparticle weight*, depends on both the sign (attractive or repulsive) and magnitude of the interactions, and its vanishing corresponds to the breakdown of the Fermi liquid description as investigated in a recent experiment at JILA^6^.

A convenient experimental technique for studying ultracold atoms is radio-frequency spectroscopy, which has been applied in many experimental as well as theoretical approaches^7^,^8^. Radio-frequency spectroscopy can, for instance, be used to obtain the *contact*^9^,^10^,^11^,^12^,^13^, a quantity describing short-range correlations in the system. By measuring momenta of the atoms transferred by the long wavelength radio-frequency field^7^,^14^,^15^, one can determine the single-particle spectral function of the atoms in the initial many-body state^15^. Furthermore, by selectively probing the system so that one considers only a particular “slice” where the density is approximately homogeneous^16^, the method allows experimental verification of theories in the unitary regime.

The theory used in this work for describing the BCS-BEC crossover is a perturbative extension of the Brueckner-Goldstone (BG) theory^17^,^18^,^19^, which has primarily been applied in the context of nuclear physics and liquid ^3^He^20^. This theory is similar to Fermi liquid theory in the sense that it has long lived quasiparticles at the Fermi surface, and an associated jump in the momentum distribution. This is in contrast to BCS theory, in which the formation of pairs results in a continuous momentum distribution. Well-formed pairs are a given in the superfluid phase of the Fermi gas, as well as in the BEC side of the BCS-BEC crossover in which two-body physics supports a (molecular) bound state. However, bound states are not always antithesis to Fermi liquid-like behaviour^21^,^22^,^23^. The goal of the present work is to study to what extent the Fermi liquid picture can be used in strongly interacting atomic gases. In particular, we describe a situation in which pairing is *not* important, and we instead focus on scattering processes between the atoms. The breakdown of the theory can then be associated with the appearance of pairs, giving physical intuition into which processes dominate the system. This can be seen as an approach complementary to BCS theory, which assumes pairs and breaks down when the pairs become unstable to decay, or as an alternative to many pseudogap theories^8^,^24^,^25^,^26^ in which noncondensed pairs are formed already at temperatures above the critical superfluid temperature.

BG theory is appealing for various reasons. It can be formulated in terms of the more well-behaved two-body scattering T-matrix, rather than the bare inter-atomic potential. Furthermore, the theory can describe the Hartree energy shift even at unitarity where the naïve (mean-field) constant energy shift 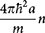 diverges as the scattering length *a* → ∞ (where *n* is the atom density and *m* is the mass of the atom). The model also provides, as a by-product, the full many-body scattering T-matrix, which, in turn, can be used for extending the model. Here we will extend the BG theory perturbatively, and use it for calculating the momentum-resolved radio-frequency response function. The perturbative processes included in the response function are shown as schematic diagrams in Fig. 1.

## Results

### Hartree shift and effective masses

The interacting two-component Fermi gas is described by the many-body Hamiltonian
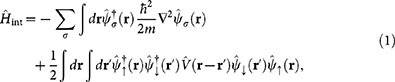
where *m* is the mass of an atom, assumed to be equal for all (pseudo)spin states, and 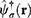 is a field operator, which annihilates (creates) an atom with (pseudo)spin *σ* ∈ {↑, ↓, *e*} at point **r**. The different components, or (pseudo)spin states, correspond to different hyperfine states of the atoms. In the presence of a magnetic field, these different internal states of the atoms are well defined with large energy gaps due to Zeeman effect. In dilute and cold atomic gases, the hyperfine states form an excellent analogy of spin-N (for bosonic atoms) or 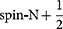 (for fermionic atoms) particles. For simplicity, we will refer to these different hyperfine states as spin-states. The atoms are assumed to be fermionic and, consequently, the field operators satisfy anticommutation relations 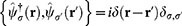. The two-particle interaction potential *V*(**r**) is assumed to be of short range, in which case its details are irrelevant. However, the two-body scattering T-matrix used below corresponds to the contact interaction pseudopotential 
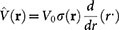
, where 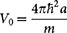 and *a* is the *s*-wave scattering length. Notice that the model involves three different hyperfine states of the atoms |↑〉, |↓〉, and |*e*〉: the initial state is a balanced mixture of |↑〉 and |↓〉 atoms, and the radio-frequency field transfers atoms from the state |↑〉 to the initially unoccupied non-interacting state |*e*〉.

The coupling with the probing radio-frequency (rf) field is described by the standard time-dependent operator in the rotating wave approximation
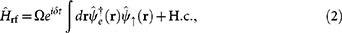
where Ω is the coupling strength and *δ* is the frequency detuning of the rf-photon from the hyperfine energy splitting between spin states |*e*〉 and |↑〉.

The transfer rate for atoms with momentum **k** in hyperfine state |↑〉 to be transferred to the |*e*〉-state at time *τ* by the rf-pulse is given by linear response theory as
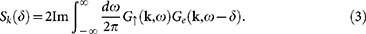
This spectral function is normalized in such a way that integration over frequency *δ* yields the occupation probability *n*(*k*) of the momentum state *k* in the initial state. Because atoms in the excited state |*e*〉 are noninteracting, and initially there are no atoms in the state, the corresponding Green's function has the simple form of a vacuum propagator
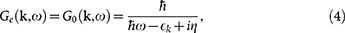
where 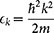 and *η* is a convergence parameter. What is needed now is the Green's function for atoms in the spin state |↑〉.

The Brueckner-Goldstone theory, outlined in the Methods section, provides a good basis for formulating a theory that can incorporate many-body interactions across the BCS-BEC crossover. The starting point is the Dyson equation, which connects the interacting Green's function and the self-energy 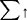:
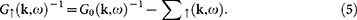
Different approximations to the self-energy then yield different many-body theories^8^,^24^,^25^,^26^,^27^,^28^,^29^,^30^,^31^. In Brueckner-Goldstone theory, one considers only self-energies on-the-energy-shell (or simply on-shell), i.e. the energy dependent part of the self-energy is neglected and evaluated at the energy equal to the interacting single-particle energy:
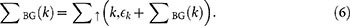
We will provide the perturbative extension of the Brueckner-Goldstone theory in a moment, but it is worthwhile to consider already the behaviour of the Brueckner-Goldstone self-energy 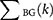 itself. It allows us to calculate several experimentally relevant quantities, such as the Hartree energy shift and effective masses.

Fig. 2 shows the calculated real part of the self-energy 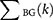 for various interaction strengths. The plot reveals the strong momentum dependence of the self-energy, particularly close to unitarity *k*_F_*a* = ±∞. The momentum dependence is easily understood^32^ when considering the two-body on-shell scattering amplitude, which for the contact interaction pseudopotential is
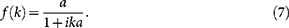
For large relative momenta 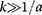, the scattering amplitude is suppressed. Hence, high momentum atoms will interact very weakly with atoms in the Fermi sea and the self-energy is suppressed. Deep inside the Fermi sea for 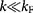, the self-energy is again suppressed. This is caused by the Pauli blocking of low-energy scattering channels due to the Fermi sea. Subsequently the self-energy has a (negative) maximum close to the Fermi surface. In the weakly interacting limit 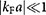, the real-part of the self-energy reproduces the usual Hartree energy shift 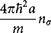, where *n_σ_* is the density of atoms in one spin state. In this limit, the momentum dependence of the scattering amplitude is also insignificant since it will not play a role until momenta 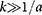.

The momentum dependence of the self-energy implies that quasiparticles behave as having an effective mass m*, which can differ from the bare atom mass *m*. The effective mass depends on momentum, and for a given momentum *k* it can be determined by fitting quadratic dispersion to the dispersion of the quasiparticle as follows
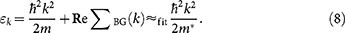
In practice, this means doing a linear, or quadratic if *k* = 0, fit to the self-energy, as exemplified in Fig. 2. In particular, the zero-momentum effective mass is
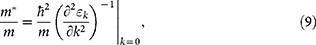
and at the Fermi momentum it is
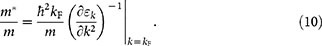
These effective masses are shown as a function of interaction strength in Fig. 3. Interestingly, the figure shows a clear maximum in the strongly interacting regime, away from unitarity. Both of the effective masses have the same qualitative behaviour, although the effect is more pronounced at the Fermi surface because interaction effects are stronger at the Fermi surface. The decreasing effective mass when crossing the unitary limit can be understood as a crossover to a repulsive single-particle branch. While the ground state in the BEC side consists of molecules, with effective mass m* = 2*m* in the far BEC limit, the unpaired fermions will in the same limit have effective mass of m* = *m*, because the single-particle branch and molecular branch become separated by a large energy gap. The present theory is unable to describe the molecular branch, but it should provide a good description of repulsively interacting unpaired fermions sufficiently far in the BEC limit.

An interesting effect is the temperature dependence of effective masses. The effective mass of zero momentum atoms increases with higher temperature while for atoms at the Fermi surface it decreases. The first effect is due to the appearance of thermal hole excitations within the Fermi sea, opening up some of the low-energy scattering channels that would otherwise have been blocked. This increases the effective interaction strength of low momentum atoms. In contrast, atoms at the Fermi surface have decreased scattering probability because the Cooper instability, which describes many-body enhancement of scattering processes around the Fermi surface, is weakened with the broadening of the Fermi surface.

Fig. 3 shows also the energy shift of zero-momentum atoms, Re Σ_BG_(0). It shows smooth behaviour near unitarity, although sufficiently deep in the BEC side the self-consistent iteration has problems finding a unique solution. Close to *k*_F_*a* ≈ 2 the model switches to the repulsive single-particle branch, involving a big change in the self-energy. While we consider this to be due to the limitations of the model, namely that it cannot simultaneously describe both the repulsive single-particle branch and the molecular branch, it is intriguing that the experiment^6^ also exhibits a sudden change to the repulsive branch at a comparable interaction strength.

### Momentum distribution, contact, and quasiparticle weight

In order to analyze momentum distributions and spectral functions, the Brueckner-Goldstone theory must be extended. Indeed, the on-shell approximation for the self-energy made in Eq. (6) yields no corrections to the non-interacting distributions. However, we can use the Dyson equation (5) for formulating a perturbative correction to the interacting Green's function as
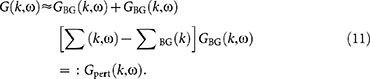
As shown in Methods section, using the perturbed Green's function *G*_pert_(**k**, *ω*) yields the momentum distribution
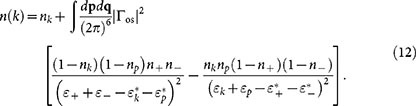
where Γ_os_ is the on-shell scattering T-matrix and the subscripts ± correspond to momenta (**k** + **p**)/2 ± **q**. The first term is simply the unperturbed occupation probability (the Fermi-Dirac distribution at fixed temperature). The other terms are the perturbative correction to the momentum distribution: the second term gives the probability that a particle has scattered to an initially empty state with momentum **k**, and the third term is the probability that an initially occupied state is empty, due to scattering to other states. The perturbative correction can be shown to conserve the number of particles, although it is not guaranteed to yield occupation numbers between 0 and 1 below the superfluid phase transition temperature. This anomalous feature is not surprising, given that we are explicitly neglecting superfluid pairing *a priori*. However, the momentum distribution is well-behaved even at unitarity when the temperature is sufficiently high (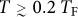). In the weakly interacting limit, Eq. (12) reproduces analytical results of Ref. 33.

Momentum distributions are plotted in Fig. 4 for various interaction strengths. The height of the momentum distribution step at the Fermi surface can be associated with the quasiparticle weight *Z*. However, at finite temperatures, thermal excitations broaden the Fermi surface, and an alternative way for characterizing *Z* is needed. We determine *Z* by calculating the largest depletion and the largest increase in the momentum distribution compared to the Fermi-Dirac distribution *n_k_*. In practice this means calculating the maximum *δn*_max_ and the minimum *δn*_min_ of the occupation number correction *δn_k_* = *n*(*k*) − *n_k_*. The quasiparticle weight *Z* is then 1 − *δn*_max_ + *δn*_min_. For a noninteracting system *Z* defined as above is equal to 1, regardless of the temperature. At zero temperature and finite interaction, *Z* is equal to the step in the momentum distribution at the Fermi surface, thus reproducing the expected behaviour of a Fermi liquid.

Fig. 5 shows these calculated quasiparticle weights as a function of interaction strength. Also plotted is an analytical zero-temperature result valid for weak repulsive interactions^17^,^33^:
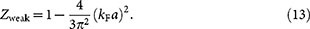
Our model reproduces this analytical result exactly in the weakly interacting limit. Our model predicts a larger quasiparticle weight at unitarity than observed in the experiment^6^. However, the theory does describe the qualitative behaviour correctly, especially that the quasiparticle weight vanishes slightly on the repulsive side of the crossover. Similar results have been obtained in Ref. 39 and one should notice that also zero-temperature mean-field BCS theory predicts the chemical potential to change sign at around *k*_F_*a* ≈ 0.55. The quasi-particle weight, particularly in the strongly interacting regime, depends rather strongly on the temperature, so the discrepancy with the experiment could partially be due to difficulties in precisely determining the temperature, but also due to the different schemes of determining the quantity *Z*.

The momentum distribution also yields the correct *k* → ∞ asymptote. For large *k* we get
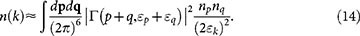
This asymptotic behaviour is clearly shown in the logarithmic plot in Fig. 4. The prefactor of the *k*^−4^ tail is called the contact parameter *C*, and from Eq. (14) we get
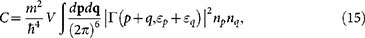
where *V* is the volume. The same result was obtained in Ref. 32 using a different approach.

Fig. 6 shows the calculated contact as a function of interaction strength for *T* = 0.2 *T*_F_ and as a function of temperature at unitarity. For weak interactions, the contact is given by the analytical result
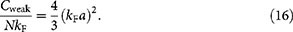
As is already well known, BCS theory is unable to reproduce this limit, but instead it predicts an exponentially decreasing contact as a function of interaction strength. The present theory reproduces the weak interaction result exactly.

The temperature dependence of the contact shows a clear maximum close to the critical temperature for superfluidity, *T*_c_ ≈ 0.2 *T*_F_, in qualitative agreement with predictions of an increase in the contact as a function of temperature for low temperatures^34^,^35^. While the present model neglects superfluid properties, it produces well-behaved results for the contact parameter even in the low temperature regime. For temperatures 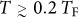 the contact decreases again. This is because the scattering T-matrix is strongly momentum dependent at unitarity, and the average relative momentum of the atoms increases with the temperature. The high-temperature limit reproduces the second order virial theorem result^36^,^37^
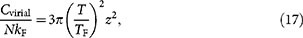
where 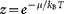.

### Momentum-resolved radio-frequency spectroscopy

The perturbative correction to the Green's function, *G*_pert_, allows also the study of momentum-resolved radio-frequency spectra. The momentum resolved spectrum, *S_k_*(*δ*), consists of a bare response and the perturbative correction. The former describes the response of the unperturbed propagator *G*_BG_:
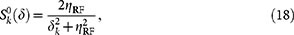
where *η*_RF_ is the linewidth of the radio-frequency field, and 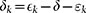. Notice, that this response already contains the momentum-dependent Hartree-type energy shift through 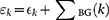.

**Figure 1:**
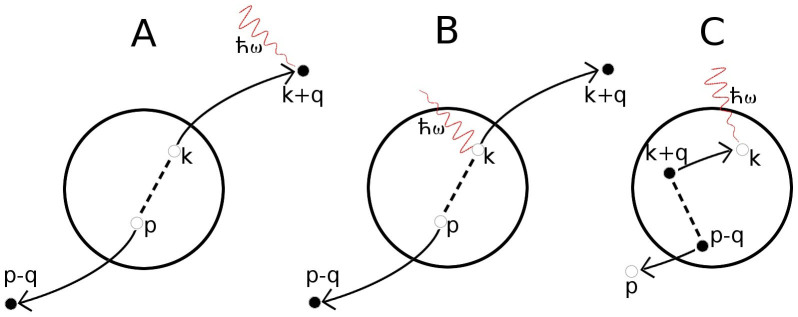
Physical scattering processes described by the perturbative corrections to the Brueckner-Goldstone response function *S*_k_(*δ*). See main text. Diagram A describes a process in which two particles with momenta + and − are scattered to momenta *k* and *p* and the radio-frequency photon of energy 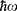 flips the spin-state of the momentum *k* atom. Diagram B describes a shadow process of diagram A, in which the atoms scatter away from states *k* and *p*, leaving holes in place. Finally, the diagram C describes a process in which the rf-photon first excites an atom with momentum *k*, leaving thus a hole in the sea of |↑〉-atoms. This is followed by the scattering of two atoms with momenta + and − into the hole in *k* and some empty state *p*.

**Figure 2:**
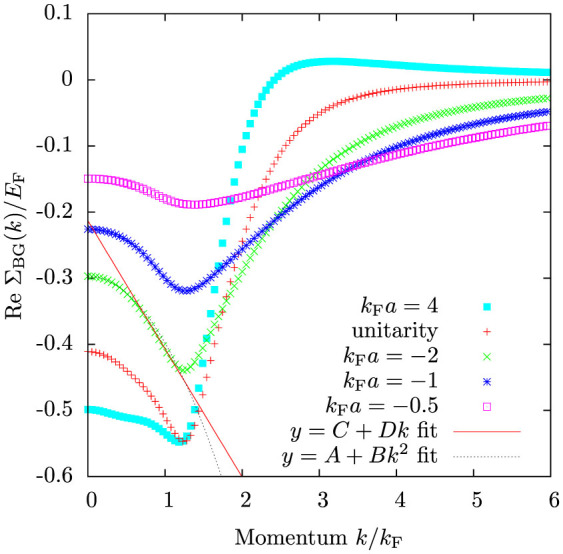
The real part of the Brueckner-Goldstone self-energy Σ_BG_ as a function of momentum for various interaction strengths. Shown are also a *y* = *A* + *Bk*^2^ fit to the *k*_F_*a* = −2 self-energy data in the range 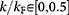 used for determining the zero momentum effective mass and a linear *y* = *C* + *Dk* fit for the data in the range 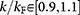 for obtaining the effective mass at the Fermi surface. Here and elsewhere, unless otherwise pointed out, the temperature is *T* = 0.2 *T*_F_ and the convergence factor *η* = 0.05 *E*_F_.

**Figure 3:**
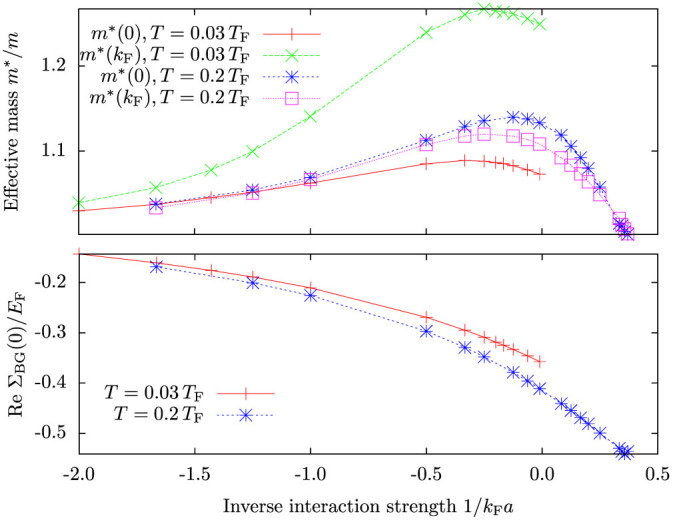
Top: the effective mass m*/*m* obtained from the Brueckner-Goldstone self-energy for zero momentum atoms and atoms at the Fermi surface. The zero momentum effective mass is obtained using a quadratic fit to the self-energy, and the effective mass at the Fermi surface using a linear fit as exemplified in Fig. 2. The *T* = 0.2 *T*_F_ data for *k* = 0 shows the error bars from the fitting. Bottom: the energy shift of zero-momentum atoms Re Σ_BG_(*k* = 0) as a function of interaction strength. Notice that the data for different temperatures has not been calculated beyond the point where the perturbative extension of the Brueckner-Goldstone model starts exhibiting nonphysical artifacts in the momentum distribution, see main text. The model works better at higher temperatures.

**Figure 4:**
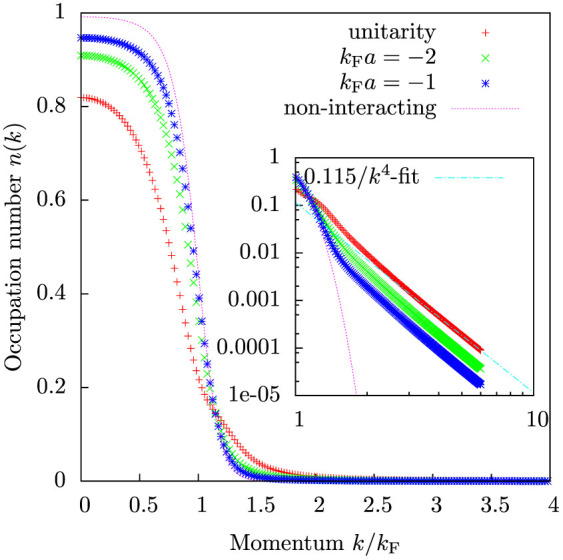
The momentum distribution from the perturbatively extended BG theory for temperature *T* = 0.2 *T*_F_. Inset shows the same data in logarithmic scale. The high momentum asymptote follows 1/*k*^4^ scaling, which is the result of short-range interactions.

**Figure 5:**
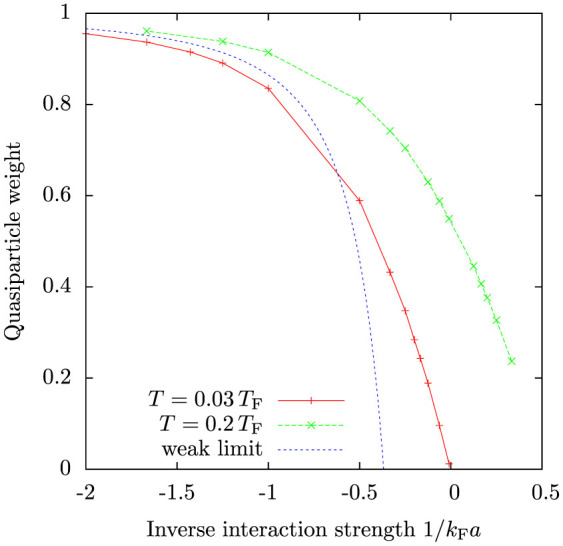
Quasiparticle weight *Z* as a function of interaction strength calculated from the momentum distributions for temperatures *T* = 0.03 *T*_F_ and *T* = 0.2 *T*_F_. Shown is also analytical result for zero temperature, valid in the weakly interacting limit.

**Figure 6:**
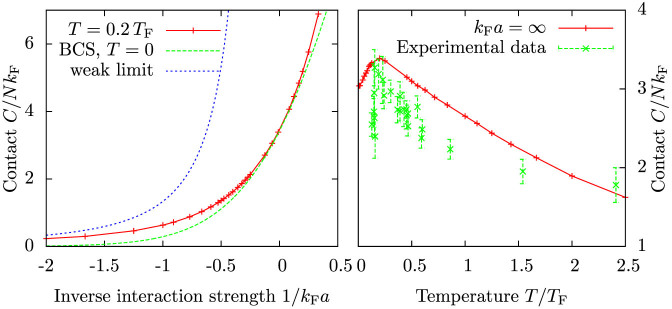
Contact as a function of interaction strength for temperature *T* = 0.2 *T*_F_, and as a function of temperature at unitarity. Shown is also the analytical result 
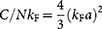
, which is valid for weak interactions. The numerical values reproduce the analytical result well at weaker interactions. Experimental data are from Ref. 16.

The perturbative correction to the response function is derived in Methods section, but it can be most easily described using schematic diagrams, shown in Fig. 1. In the diagram A, before the absorption of the photon, the particles in the scattered states are simple virtual excitations with the energy of the scattered state being equal to the initial energy of the + and −-atoms. In order for the rf-photon to be absorbed, and the *k* momentum atom being transferred to the excited |*e*〉-spin state, the photon will need to supply the required energy to make the virtual state real. Hence the process is on-resonance at frequency 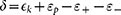, corresponding to the increase in the kinetic energies due to the scattering, Δ*E* = *ε_p_* + *ε_k_* − *ε*_+_ − *ε_−_*, and the energy change due to the absorption of the radio-frequency photon 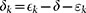.

If there is a possibility of finding atoms at high momentum states, as described by the diagram A, the probability of finding atoms in low momentum states must decrease. This is indeed the effect of the diagram B in Fig. 1. The diagram provides a spectral response which has the same overall functional form as the bare response, 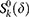, and since it describes a vacancy, it has the opposite sign.

The process described in the diagram C involves dynamics generated by the creation of the hole excitation^38^, and it does not influence ground state properties, such as the momentum distribution.

**Figure 7:**
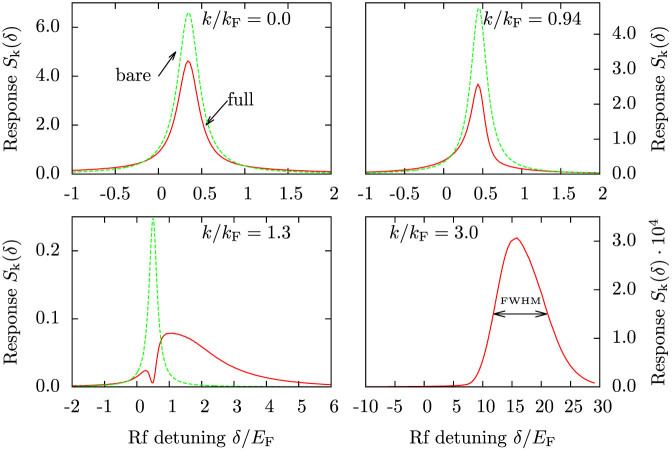
Momentum-resolved radio-frequency spectral functions *S_k_*(*δ*) for atoms with different momenta *k*. Top figures: for low momenta *k* < *k*_F_ the full response exhibits a significantly weaker response than the bare response, which involves only the unperturbed Brueckner-Goldstone contribution (Hartree shift). The corrections do not cause any additional shift but lower the peak and create a very broad incoherent background response (stronger tails). Bottom figures: for larger momenta *k* > *k*_F_, the bare response vanishes rapidly as only thermal quasiparticle excitations contribute to the unperturbed response. However, the full response acquires a very broad asymmetric peak, corresponding to scattered atoms. The calculated width (FWHM) of the spectral peak shown for *k* = 3 *k*_F_ data is 9.2 *E*_F_. Notice the additional factor 10^4^ included in the *k* = 3 *k*_F_ plot. Here, and in all the response data, the interaction strength is *k*_F_*a* = −4 and temperature *T* = 0.2 *T*_F_.

Fig. 7 shows the momentum-resolved spectra calculated for different momenta. For hole excitations, *k* < *k*_F_, the second-order correction to the spectrum lowers the Lorentzian bare response peak significantly and creates a broad background response. Due to the weakness of the background response, the full width at half maximum (FWHM) of the full response is unaffected by the corrections.

The broad background response originates from the diagram C in Fig. 1. The scattering of the hole can decrease or increase the energy of the atoms, thus providing a resonant total process at energies significantly away from the single-particle resonance. Also the diagram B in Fig. 1 affects low momentum atoms. However, since the contribution has exactly the same lineshape as the bare response, it can only lower the spectral peak by the amount corresponding to the quantum depletion of the momentum distribution.

Fig. 7 shows also the momentum-resolved spectrum for an atom with momentum *k* = 1.3 *k*_F_. At this momentum, there are still some thermal quasiparticle excitations, and consequently the bare response still appears. In the full response, this quasiparticle peak is broadened and lowered, but in addition there appears a very broad and highly asymmetric feature. For even higher momentum, *k* = 3 *k*_F_, the bare response is completely absent, since the momentum state is not populated in the unperturbed state (the Fermi-Dirac occupation probability is vanishingly low). However, the full response still exhibits a very broad spectral peak. The response, and the broad feature in the *k* = 1.3 *k*_F_ data, comes from the diagram A in Fig. 1. The radio-frequency field will need to supply the required energy to make the virtual excitation real. However, since the transferred atom with momentum **k** may have reached the scattering state through interaction with any of the atoms in the |↓〉-Fermi sea, the virtual state has a very broad energy spectrum.

**Figure 8:**
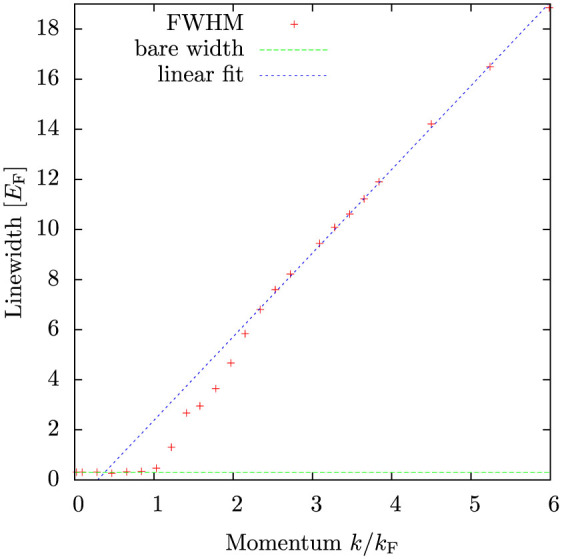
The widths (full width at half-maximum) of the momentum-resolved spectra as a function of momentum. At low momenta, the width is dominated by the constant linewidth 2*η* = 0.3 *E*_F_ of the bare response (the linewidth of single-particle excitations 0.05 *E*_F_ and the linewidth of the probing field 0.1 *E*_F_ provide the total linewidth *η* = 0.15 *E*_F_), but at high momenta the width increases linearly with momentum. The linear fit has slope 3.34 *E*_F_/*k*_F_ and is calculated for data 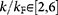. Here *k*_F_*a* = −4 and temperature *T* = 0.2 *T*_F_.

Fig. 8 shows the full-width at half-maximum (FWHM) of the spectral peaks as a function of momentum *k*. While FWHM is too rough a measure for revealing the effect of the hole dynamics at low momenta, which produces the broad incoherent background response seen in low momentum data in Fig. 7, it does show a linear increase of the spectral width at high momenta. The increase is in drastic contrast with the width of the spectral peak predicted by BCS theory, which yields a spectral width limited only by the linewidth of the radio-frequency field.

If one interprets the results as a signature of bound pairs, the width of the peak can be understood as a measure of the imprecision in the center-of-mass momenta of pairs. Indeed, consider a bound pair of center-of-mass momentum *q*. It can be described by the pair creation operator
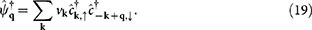
Performing momentum-resolved spectroscopy for a pair created by such operator yields the momentum-resolved spectral function
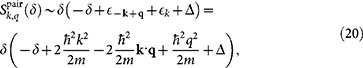
where Δ is the pair binding energy (the initial energy of the pair). The spectral function is thus a narrow peak at frequency 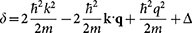.

If there is spread in the center-of-mass momenta of the pairs, the spectral peak becomes broader. For example, if pairs have center-of-mass momenta in the interval 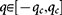, the width of the spectral function is
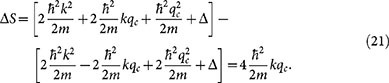
The width of the observed spectrum is thus a function that increases linearly with the momentum *k* and the slope is given by the center-of-mass momentum spread of the pairs.

Considering the widths in Fig. 8, the corresponding pair center-of-mass momenta would be of the order of the Fermi momentum: a linear fit to data in the interval *k*/*k*_F_ ∈ [2, 6] gives a slope of 3.34, translating into a center-of-mass momentum width *q_c_* ≈ 0.84 *k*_F_. This slope can be compared with the fitted pair temperature *T*_P_ observed in the experiment^6^, which is approximately *T*_P_ ≈ 0.8 *T*_F_ throughout the BCS-BEC crossover. The observed pair temperature appears unrelated to the actual gas temperature *T* ≈ 0.25 *T*_F_. This may be a sign that the bosonic pairs are not thermalized with the rest of the Fermi gas.

Since the present theory can describe the observed pair temperature without including bound pairs in the theoretical description^39^, one can ask how actual condensed pairs would show up in the spectrum. Considering the condensation of zero-momentum Cooper pairs in the superfluid phase, we expect to observe a narrow spectral feature in the high momentum momentum-resolved spectrum *S_k_*(*δ*) when the temperature is reduced below the critical temperature.

**Figure 9:**
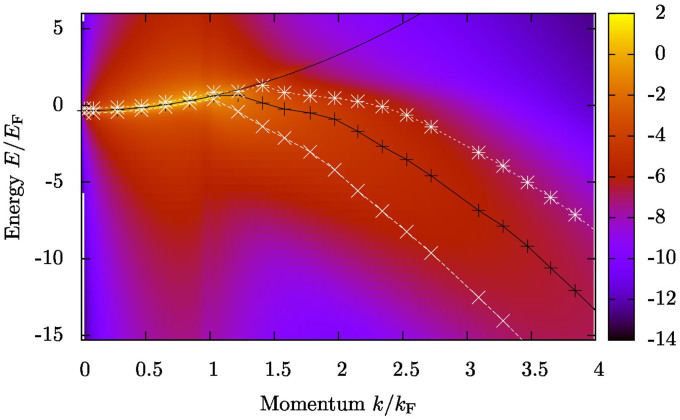
Momentum-resolved radio-frequency spectra *k*^2^*S_k_*(*δ*). The *k*^2^-prefactor provides the effect of the density of states, to provide easier comparison with experimental data. The colour bar shows the magnitude of the response in a logarithmic scale. Shown are also the position of the spectral maximum (black crosses) and the frequencies at which the response is half of the maximum value (white symbols) – the full width at half-maximum is then the energy separation of the two half-maximum energies, used in Fig. 8. The solid black line is a quadratic fit to the quasiparticle branch 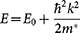, with *E*_0_ = −0.33 *E*_F_ and m* = 1.1 *m*. Here *k*_F_*a* = −4 and temperature *T* = 0.2 *T*_F_.

Fig. 9 shows the full momentum resolved rf-spectrum. At low momenta *k* < *k*_F_ the response has quite a narrow linewidth, but at higher momenta a broad back-bending branch appears^40^. Thermal excitations show up as a narrow quasiparticle branch extending beyond momenta *k* > *k*_F_. Notice, that many pseudogap theories^41^,^42^,^43^,^44^, exhibit an additional spectral branch at low momenta *k* < *k*_F_ and at positive energies *E* > 0. This branch is a remnant of the thermally excited quasiparticle branch present already in BCS theory but also in the fully self-consistent field theory in the superfluid phase^45^. It is noteworthy that the branch is missing in the present theory, but it is also missing from the experimental spectra^6^.

The momentum resolved spectrum is more sensitive than the momentum distribution to the perturbative corrections. Indeed, the momentum distribution is well behaved across the BCS-BEC crossover for sufficiently high temperatures. In contrast, the momentum resolved spectrum has artifacts near the Fermi surface, such as areas where the response becomes negative. This happens when the perturbative correction becomes larger than the unperturbed value, signaling a breakdown of the perturbative approach. The reason these artifacts do not appear in momentum distribution is that the perturbative correction terms partially cancel each other. However, since the different processes (or diagrams) in the perturbative correction are resonant at different energies, the partial cancellation does not happen when the energies are resolved, such as in the response function. We are thus limited to weaker interactions in the momentum-resolved spectroscopy.

**Figure 10:**
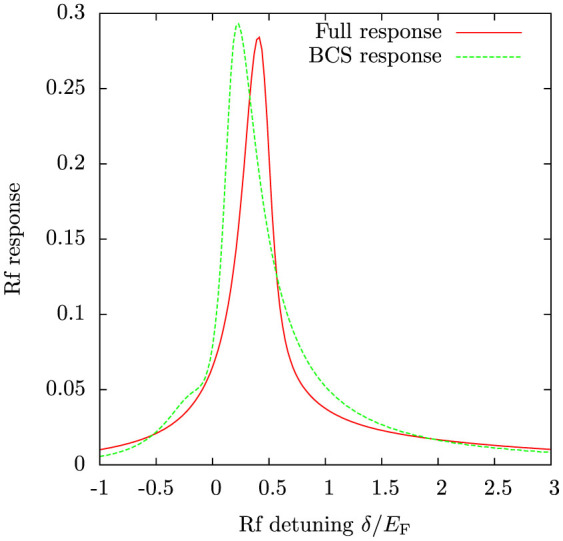
Radio-frequency spectrum obtained by integrating the momentum resolved spectra over momentum *k*. Shown is also the spectrum obtained from BCS theory. The main differences between the two spectra are the slightly more asymmetrical lineshape and a broad bump at negative detuning *δ* ≈ −0.3 *E*_F_ in the BCS response. The latter feature comes from thermal quasiparticle excitations present in the BCS theory^59^. However, the uniform rf-response is expected to have only a single peak^60^. Here *k*_F_*a* = −4 and temperature *T* = 0.2 *T*_F_.

Fig. 10 shows the calculated (non-momentum-resolved) radio-frequency spectrum and the corresponding result from BCS theory, obtained from the momentum-resolved spectrum by integrating over the momentum *k*. Even though the two theories yield qualitatively different momentum-resolved spectra, the two agree surprisingly well in the integrated response. The interpretations of the two spectra in Fig. 10 are, however, quite different. While the energy shift in the BCS spectrum is due to pair binding energy Δ, the present theory explains it as a simple Hartree-type energy shift. It thus appears that the Hartree energy shift turns into pair binding energy^46^,^47^ when the transition from the normal phase to the superfluid phase occurs. The Hartree shift is also the dominant energy shift in the weakly attractive regime, even at zero temperature^45^.

The radio-frequency spectrum in Fig. 10 is in good qualitative agreement with experimental spectra for uniform systems^16^,^48^. It will be very interesting to see how the present theory works with spin-imbalanced systems, and, particularly, whether the model can produce a double peak structure as observed in Ref. 49. However, this goes beyond the scope of the present investigation.

## Discussion

In conclusion, we have perturbatively extended the Brueckner-Goldstone (BG) theory and applied it to a strongly interacting Fermi gas in the BCS-BEC crossover. The theory provides direct access to momentum distributions and momentum-resolved radio-frequency spectra. The momentum distributions are consistent with exact asymptotic results from the Tan relations, giving a high-momentum tail with an algebraic 1/*k*^4^-decay. Moreover, the strength of the algebraic decay is in good agreement with experimentally determined values. We also find good agreement between the radio-frequency spectra predicted from the extended BG theory and experimental spectra. Furthermore, we predict the breakdown of Fermi liquid behaviour at finite repulsion, also in agreement with the JILA experiment^6^.

The model used here neglected possible bound pairs in order to help formulate a theory in line with Fermi liquid theory. However, it is important to notice that the model does not exclude pair correlations. Indeed, the perturbative correction to Brueckner-Goldstone theory can be understood as introducing pair correlations, that were lost in the on-shell approximation of the self-energy. Many properties of the model studied here, particularly the back-bending part of the high momentum momentum resolved spectrum, can be understood as a signature of these correlations^40^. But pair correlations are unavoidable in interacting systems, and have very little to do with presence of bound pairs.

At low temperatures and strong interactions the perturbative model breaks down and produces unphysical features in some quantities. Particularly the momentum resolved spectral function near the Fermi surface is sensitive to the perturbation. The quasiparticle weight is less sensitive due to destructive interference between different terms in the perturbative correction. These problems are of course typical of perturbative theories when corrections become large. However, the unperturbed Brueckner-Goldstone model itself is well behaved across the BCS-BEC crossover. The model may thus provide a good basis for a non-self-consistent extension, in the spirit of the Nozières-Schmitt-Rink theory^54^. Still, the results presented here should provide a qualitatively correct picture at temperatures above the critical superfluid temperature *T*_c_, but with the understanding that a nonperturbative treatment would renormalize corrections.

The present work points out several quantities that could be studied in the experiments, such as the broad incoherent background response in the momentum-resolved rf-spectrum at low momenta, the asymmetry of the spectral linewidth, and the linear scaling of the width of the high momentum response peak. Furthermore, we expect the transition to the supefluid state, with condensed pairs, to be reflected as a narrowing or at least as an emergence of some narrow feature in the high momentum radio-frequency response.

## Methods

In this section we will first present the self-consistent Brueckner-Goldstone theory and then explain how it is extended using perturbation theory. The result is a theory that can describe Hartree-type energy shifts (although momentum dependent) and predicts qualitatively correct behaviour for the asymptotic momentum distribution. In the weakly interacting limit, the theory reproduces well-known analytical results, but the model is well-behaved also across the BCS-BEC crossover.

For a spin-balanced system and equal masses, the Green's functions and self-energies for both |↑〉- and |↓〉-spin states are identical. Hence for simplicity, we will consider below only the |↑〉 spin state.

The many-body (dressed) Green's function can be calculated from the Dyson equation (using the four-vector notation *K* = (**k**, *ω*))
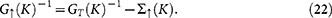
The non-interacting finite temperature Green's function at temperature *T* is given by 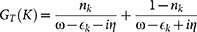, where 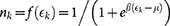 is the Fermi-Dirac distribution, *μ* is the chemical potential and *β* = 1/*k_B_T*. This non-interacting Green's function *G_T_*(*K*) describes both hole (first term) and particle (second term) excitations in the thermal Fermi sea, but neglects any interaction effects. These effects enter the dressed Green's function *G*_↑_(*K*) through the self-energy Σ_↑_(*K*), which in the ladder approximation is
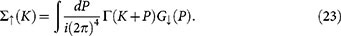
Here Σ(*K*) is the many-body scattering T-matrix
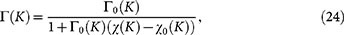
where the pair susceptibility 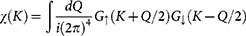 and the two-body scattering T-matrix^50^ 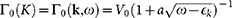. In order to avoid double counting certain scattering terms, one needs to remove the vacuum pair susceptibility 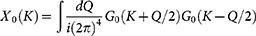 from the pair susceptibility *χ*(*K*).

In the Brueckner-Goldstone theory, the frequency dependence of the self-energy is neglected and the value of the self-energy is evaluated on-shell. That is, the self-energy entering the Brueckner-Goldstone Green's function *G*_BG_(*K*)^−1^ = *G_T_* (*K*)^−1^ − Σ_BG_(*k*) is solved iteratively as
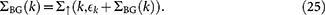
The theory is fully self-consistent, in the sense that the Brueckner-Goldstone Green's function *G*_BG_(*K*), obtained from the Dyson equation, is used in the pair susceptibility *χ*(*K*) and the self-energy Σ_↑_(*K*).

Due to the simplicity of the self-energy, the Brueckner-Goldstone Green's function also has a very simple form at finite temperatures
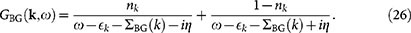
Notice that the Brueckner-Goldstone self-energy will not affect the momentum distribution and thus it is sufficient to solve the distribution *n_k_* for the noninteracting system when fixing the number of atoms in the system.

The real part of the Brueckner-Goldstone self-energy Re Σ_BG_(*k*) can be interpreted as the Hartree energy shift since in the weakly interacting 3d limit it yields the standard result 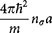, where *n_σ_* is the atom density in spin state |*σ*〉. However, the energy shift depends on the momentum because of the momentum dependence of the scattering T-matrix. The imaginary part Im Σ_BG_(*k*) has correct Fermi liquid features so that for *k* > *k*_F_ the imaginary part is negative, corresponding to particle excitations, and for *k* < *k*_F_ the imaginary part is positive as required for hole excitations. At the Fermi surface the imaginary part vanishes, which is a signature that the Brueckner-Goldstone theory provides well-defined quasiparticles.

While the Brueckner-Goldstone theory is self-consistent, it is unable to describe pairs. The pair formation is caused by the presence of poles in the scattering T-matrix, and it appears in the self-energy landscape Σ(*k*, *ω*) as a peak along the 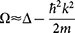-branch, where Δ is the pair binding energy. This branch is missed by the Brueckner-Goldstone self-energy Σ_BG_. Since the pair formation cannot therefore be self-consistently described, we make a further approximation and neglect poles in the many-body scattering T-matrix. In practice, this is performed by replacing the many-body scattering T-matrix by the on-shell T-matrix. The Brueckner-Goldstone self-energy now acquires a particularly simple form:
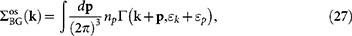
where 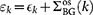.

Neglecting the poles of the scattering T-matrix Γ, however, breaks the analytical structure of the equation and results in unphysical functional dependence of the imaginary part of the self-energy. In particular, the imaginary part of the self-energy no longer changes sign at the Fermi surface^32^. To avoid the problem, we neglect the imaginary part obtained from the Brueckner-Goldstone self-energy altogether and instead use a fixed imaginary part. While this means that quasiparticle excitations at the Fermi surface still have a finite lifetime, we have checked that it does not have any qualitative effect in the results shown below. However, while the value of *η* in the vicinity of the Fermi surface is not important, the overall value of *η* does affect the results to some extent. Throughout this work, we use the value *η* = 0.05*E*_F_, corresponding roughly to the imaginary part of the Brueckner-Goldstone self-energy at zero momentum for interaction strength *k*_F_*a* = −2, i.e. *η* = Im Σ_BG_(*k* = 0). While different choices of parameter *η* do not result in any qualitative changes, the actual numerical values of the contact, effective mass and quasiparticle ratio change by at most 10% when *η* is decreased by factor 0.5 or increased by factor 1.5.

The self-consistent Brueckner-Goldstone self-energy Σ_BG_(*k*), and the associated Green's function *G*_BG_(*K*), provide a good basis for a perturbative expansion. Indeed, as shown analytically in Ref. 51, the expansion done in Eq. (11) satisfies the Migdal-Luttinger theorem^52^, yielding a step in the zero-temperature momentum distribution at the Fermi surface and even satisfying number conservation. Furthermore, the expansion allows calculating values of many physical observables, such as the momentum-resolved radio-frequency spectrum.

The spectrum is defined in Eq. (3). Using the perturbed Green's function defined in Eq. (11) we get
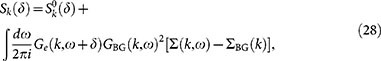
where 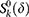 is the bare response in Eq. (18) and the self-energy is defined in Eq. (23). The self-energy contains the many-body scattering T-matrix Γ = Γ(**k** + **p**, *ω* + Ω), which can be expressed in terms of the on-shell T-matrix Γ_os_. In the on-shell T-matrix, the frequencies *ω* and Ω are replaced by the energies *ε_k_* and *ε_p_* of the incoming (scattering) particles. The many-body scattering T-matrix can now be written as
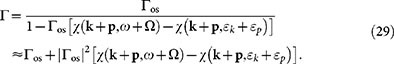
The response Eq. (28) becomes now
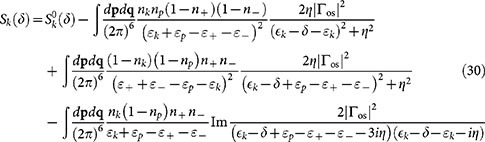
where ± indices refer to momenta (**k** + **p**)/2 ± **q**.

From the spectrum, we can obtain also the momentum distribution by integrating over detuning *δ*. Simple algebra leads into Eq. (12). The same equation was obtained also in Ref. 51 but there the many-body scattering T-matrix Γ was the Brueckner's reaction matrix and the single-particle energies neglected the self-energy shift. The two correction terms to the momentum distribution cancel each other when integrated over the momentum *k*, satisfying thus the number conservation.

To conclude this section, we would like to give a brief comparison with other many-body theories used for describing strongly interacting dilute Fermi gases. The various theories differ by the level of self-consistency and they can be divided in groups based on whether the various Green's functions in the self-energy (23) and the T-matrix (24) are dressed *G*(*K*) or bare *G*_0_(*K*)^53^. Hence we have non-self-consistent theories^30^,^44^,^54^, partly self-consistent theory^41^ and even a fully self-consistent theory^45^ This categorization does not fully do justice to the differences between various theories, as there are also other differences, for example truncation of the Dyson's equation (5)^54^,^55^, decomposition of the T-matrix^41^, and the use of separable potentials^44^. Furthermore, the list is not exhaustive as also virial (for review, see Ref. 37) and T-matrix theories^40^ have been used for describing momentum-resolved spectroscopy experiments.

The key features of the present theory are the self-consistency of the self-energy iteration and the use of on-shell energies in the self-energy. The dressed Green's function is used both in the many-body scattering T-matrix and in the self-energy. However, despite being fully self-consistent in the sense of the above categorization, the theory is far simpler than the fully self-consistent theory of Ref. 45. Neglecting the frequency dependence of the self-energy, Eq. (25), makes the theory quite different from all above theories. At the level of the first iteration of the self-energy, the present model yields *G*_0_*G*_0_*G*_0_ theories, except for the approximation in which poles of the many-body scattering T-matrix are neglected. While the approximation is hardly justifiable at low temperatures, the self-consistent iteration of the self-energy actually lowers the peaks in the T-matrix landscape. In addition, corrections to the momentum distribution will further weaken the many-body pair formation. While the latter effect was not included in the present work, its effect was considered in Ref. 32.

Assessing the validity of the various theories is generally difficult from within the theory. Most of the theories can describe well the various limits, such as the high temperature limit and weakly interacting systems, considered also here. The polaron problem is another good benchmark for theories. The Brueckner-Goldstone model was compared with the variational ansatz^56^ in the case of 1d^57^ and 3d polarons^32^. As is now well known^58^, all self-consistent theories have difficulties in describing the polaron problem due to the importance of destructive interference between various diagrams.

## References

[b1] Mirzaei S. I. *et al.* Spectroscopic evidence for Fermi liquid-like energy and temperature dependence of the relaxation rate in the pseudogap phase of the cuprates. Proc. Nat. Acad. Sci. USA 110, 5774–5778 (2013). 23536291 10.1073/pnas.1218846110 PMC3625325

[b2] Randeria M. & Taylor E. Crossover from Bardeen-Cooper-Schrieffer to Bose-Einstein condensation and the unitary Fermi gas. Annu. Rev. Condens. Matter Phys. 5, 209–232 (2014).

[b3] Thomas J. Unitary Fermi gases. Levin K., , Fetter A. L., & Stamper-Kurn D. M., eds. (eds.) Ultracold Bosonic and Fermionic Gases. 157–175 (Elsevier, 2012).

[b4] Kokkelmans S. J. J. M. F. Feshbach resonances in ultracold gases. Törmä P., & Sengstock K., eds. (eds.) Quantum gas experiments - exploring many-body states. (Imperial College Press, London, 2014).

[b5] Nascimbène S. *et al.* Fermi-liquid behaviour of the normal phase of a strongly interacting gas of cold atoms. Phys. Rev. Lett. 106, 215303 (2011). 21699311 10.1103/PhysRevLett.106.215303

[b6] Sagi Y., Drake T. E., Paudel R., Chapurin R. & Jin D. S. Breakdown of Fermi liquid description for strongly interacting fermions. arXiv, 1409.4743 (2014). 10.1103/PhysRevLett.114.075301 25763961

[b7] Chen Q., He Y., Chien C.-C. & Levin K. Theory of radio-frequency spectroscopy experiments in ultracold Fermi gases and their relation to photoemission in the cuprates. Rep. Prog. Phys. 72, 122501 (2009).

[b8] Levin K. & Hulet R. The Fermi gases and superfluids: experiment and theory. Levin K., , Fetter A. L., & Stamper-Kurn D. M., eds. (eds.) Ultracold Bosonic and Fermionic Gases. 69–94 (Elsevier, 2012).

[b9] Tan S. Energetics of a strongly correlated Fermi gas. Ann. Phys. 323, 2952 (2008).

[b10] Tan S. Large momentum part of a strongly correlated Fermi gas. Ann. Phys. 323, 2971 (2008).

[b11] Tan S. Generalized virial theorem and pressure relation for a strongly correlated Fermi gas. Ann. Phys. 323, 2987 (2008).

[b12] Braaten E. The BCS-BEC crossover and the unitary Fermi gas. Zwerger W., ed. (ed.) The BCS-BEC Crossover and the Unitary Fermi Gas (Springer, Heidelberg, 2012).

[b13] Stewart J. T., Gaebler J. P., Drake T. E. & Jin D. S. Verification of universal relations in a strongly interacting Fermi gas. Phys. Rev. Lett. 104, 235301 (2010). 20867250 10.1103/PhysRevLett.104.235301

[b14] Stewart J. T., Gaebler J. P. & Jin D. S. Using photoemission spectroscopy to probe a strongly interacting Fermi gas. Nature 454, 744 (2008). 18685703 10.1038/nature07172

[b15] Gaebler J. P. *et al.* Observation of pseudogap behaviour in a strongly interacting Fermi gas. Nat. Phys. 6, 569 (2010).

[b16] Sagi Y., Drake T. E., Paudel R. & Jin D. S. Measurement of the homogeneous contact of a unitary Fermi gas. Phys. Rev. Lett. 109, 220402 (2012). 23368108 10.1103/PhysRevLett.109.220402

[b17] Fetter A. L. & Walecka J. D. Quantum theory of many-particle systems (McGraw-Hill, New York, 1971).

[b18] Brueckner K. A. & Levinson C. A. Approximate reduction of the many-body problem for strongly interacting particles to a problem of self-consistent fields. Phys. Rev. 97, 1344–1352 (1955).

[b19] Goldstone J. Derivation of the Brueckner many-body theory. Proc. R. Soc. A. 239, 267–279 (1957).

[b20] Glyde H. R. & Hernadi S. I. Effective interactions in liquid ^3^He. Phys. Rev. B 28, 141 (1983).

[b21] Engelbrecht J. R. & Randeria M. Low-density repulsive Fermi gas in two dimensions: Bound-pair excitations and Fermi-liquid behaviour. Phys. Rev. B 45, 12419 (1992). 10.1103/physrevb.45.12419 10001279

[b22] Cazalilla M. A. A composite fermion approach to the ultracold dilute Fermi gas. Int. J. Mod. Phys. B 25, 329 (2011).

[b23] Koschorreck M. *et al.* Attractive and repulsive Fermi polarons in two dimensions. Nature 485, 619 (2012). 22660322 10.1038/nature11151

[b24] Perali A., Pieri P., Strinati G. C. & Castellani C. Pseudogap and spectral function from superconducting fluctuations to the bosonic limit. Phys. Rev. B 66, 024510 (2002).

[b25] Hu H., Liu X.-J., Drummond P. D. & Dong H. Pseudogap pairing in ultracold Fermi atoms. Phys. Rev. Lett. 104, 240407 (2010). 20867287 10.1103/PhysRevLett.104.240407

[b26] Wlazłowski G., Magierski P., Drut J. E., Bulgac A. & Roche K. J. Cooper pairing above the critical temperature in a unitary Fermi gas. Phys. Rev. Lett. 110, 090401 (2013). 23496691 10.1103/PhysRevLett.110.090401

[b27] Haussmann R. Crossover from BCS superconductivity to Bose-Einstein condensation: A self-consistent theory. Z. Phys. B 91, 291 (1993).

[b28] Haussmann R. Properties of a Fermi liquid at the superfluid transition in the crossover region between BCS superconductivity and Bose-Einstein condensation. Phys. Rev. B 49, 12975 (1994). 10.1103/physrevb.49.12975 10010209

[b29] Haussmann R., Rantner W., Cerrito S. & Zwerger W. Thermodynamics of the BCS-BEC crossover. Phys. Rev. A 75, 023610 (2007).

[b30] Watanabe R., Tsuchiya S. & Ohashi Y. Superfluid density of states and pseudogap phenomenon in the BCS-BEC crossover regime of a superfluid Fermi gas. Phys. Rev. A 82, 043630 (2010).

[b31] Gubbels K. B. & Stoof H. T. C. Interacting preformed Cooper pairs in resonant Fermi gases. Phys. Rev. A 84, 013610 (2011).

[b32] Kinnunen J. J. Hartree shift in unitary Fermi gases. Phys. Rev. A 85, 012701 (2012).

[b33] Sartor R. & Mahaux C. Self-energy, momentum distribution, and effective masses of a dilute Fermi gas. Phys. Rev. C 21, 1546 (1980).

[b34] Drut J. E., Lähde T. A. & Ten T. Momentum distribution and contact of the unitary Fermi gas. Phys. Rev. Lett. 106, 205302 (2011). 21668239 10.1103/PhysRevLett.106.205302

[b35] Yan Y. & Blume D. Harmonically trapped Fermi gas: Temperature dependence of the Tan contact. Phys. Rev. A 88, 023616 (2013).

[b36] Hu H., Liu X.-J. & Drummond P. D. Universal thermodynamics of a strongly interacting Fermi gas: theory versus experiment. New J. Phys. 12, 063038; 10.1088/1367-2630/12/6/063038 (2010).

[b37] Liu X.-J. Virial expansion for a strongly correlated Fermi system and its application to ultracold atomic Fermi gases. Phys. Rep. 524, 37 (2013).

[b38] Leskinen M. J., Kajala J. & Kinnunen J. J. Resonant scattering effect in spectroscopies of interacting atomic gases. New J. Phys. 12, 083041; 10.1088/1367-2630/12/8/083041 (2010).

[b39] Perali A. *et al.* Photoemission spectrum and effect of inhomogeneous pairing fluctuations in the BCS-BEC crossover regime of an ultracold Fermi gas. Phys. Rev. Lett. 106, 060402 (2011).21405446

[b40] Schneider W. & Randeria M. Universal short-distance structure of the single-particle spectral function of dilute Fermi gases. Phys. Rev. A 81, 021601 (2010).

[b41] Chen Q. & Levin K. Momentum resolved radio frequency spectroscopy in trapped Fermi gases. Phys. Rev. Lett. 102, 190402 (2009). 19518929 10.1103/PhysRevLett.102.190402

[b42] Magierski P., Wlazłowski G., Bulgac A. & Drut J. E. Finite-temperature pairing gap of a unitary Fermi gas by quantum Monte Carlo calculations. Phys. Rev. Lett. 103, 210403 (2009). 20366021 10.1103/PhysRevLett.103.210403

[b43] Tsuchiya S., Watanabe R. & Ohashi Y. Photoemission spectrum and effect of inhomo-geneous pairing fluctuations in the BCS-BEC crossover regime of an ultracold Fermi gas. Phys. Rev. A 82, 033629 (2010).

[b44] Palestini F., Perali A., Pieri P. & Strinati G. C. Dispersions, weights, and widths of the single-particle spectral function in the normal phase of a Fermi gas. Phys. Rev. B 85, 024517 (2012).

[b45] Haussmann R., Punk M. & Zwerger W. Spectral functions and rf response of ultracold fermionic atoms. Phys. Rev. A 80, 063612 (2009).

[b46] Chin C. *et al.* Observation of the pairing gap in a strongly interacting Fermi gas. Science 305, 1128 (2004). 15272125 10.1126/science.1100818

[b47] Kinnunen J. J., Rodríguez M. & Törmä P. Pairing gap and in-gap excitations in trapped fermionic superfluids. Science 305, 1131 (2004). 15272124 10.1126/science.1100782

[b48] Shin Y.-i., Schunck C. H., Schirotzek A. & Ketterle W. Tomographic rf spectroscopy of a trapped Fermi gas at unitarity. Phys. Rev. Lett. 99, 090403 (2007). 17930995 10.1103/PhysRevLett.99.090403

[b49] Schirotzek A., Shin Y.-i., Schunck C. H. & Ketterle W. Determination of the superfluid gap in atomic Fermi gases by quasiparticle spectroscopy. Phys. Rev. Lett. 101, 140403 (2008). 18851509 10.1103/PhysRevLett.101.140403

[b50] Morgan S. A., Lee M. D. & Burnett K. Off-shell *T* matrices in one, two, and three dimensions. Phys. Rev. A 65, 022706 (2002).

[b51] Mahaux C. & Sartor R. Theoretical approaches to the momentum distribution of a normal Fermi liquid. Phys. Rep. 211, 53 (1992).

[b52] Migdal A. B. Theory of Finite Fermi Systems and Applications to Atomic Nuclei (Interscience( Wiley), New York, 1967).

[b53] Hu H., Liu X.-J. & Drummond P. D. Comparative study of strong-coupling theories of a trapped Fermi gas at unitarity. Phys. Rev. A 77, 061605(R) (2008).

[b54] Nozières P. & Schmitt-Rink S. Bose condensation in an attractive fermion gas: From weak to strong coupling superconductivity. J. Low Temp. Phys. 59, 195 (1985).

[b55] Levin K., Chen Q., Chien C.-C. & He Y. Comparison of different pairing fluctuation approaches to BCS-BEC crossover. Ann. Phys. 325, 233 (2010).

[b56] Chevy F. Universal phase diagram of a strongly interacting Fermi gas with unbalanced spin populations. Phys. Rev. A 74, 063628 (2006). 10.1103/PhysRevLett.96.130401 16711969

[b57] Doggen E. V. H. & Kinnunen J. J. Energy and contact of the one-dimensional Fermi polaron at zero and finite temperature. Phys. Rev. Lett. 111, 025302 (2013). 23889413 10.1103/PhysRevLett.111.025302

[b58] Combescot R. & Giraud S. Normal state of highly polarized Fermi gases: full many-body treatment. Phys. Rev. Lett. 101, 050404 (2008). 18764378 10.1103/PhysRevLett.101.050404

[b59] Kinnunen J. J., Rodríguez M. & Törmä P. Signatures of superfluidity for Feshbach-resonant Fermi gases. Phys. Rev. Lett. 92, 230403 (2004). 15245145 10.1103/PhysRevLett.92.230403

[b60] Massignan P., Bruun G. M. & Stoof H. T. C. Twin peaks in rf spectra of Fermi gases at unitarity. Phys. Rev. A 77, 031601 (2008).

